# A mesoporous cationic thorium-organic framework that rapidly traps anionic persistent organic pollutants

**DOI:** 10.1038/s41467-017-01208-w

**Published:** 2017-11-07

**Authors:** Yuxiang Li, Zaixing Yang, Yanlong Wang, Zhuanling Bai, Tao Zheng, Xing Dai, Shengtang Liu, Daxiang Gui, Wei Liu, Meng Chen, Lanhua Chen, Juan Diwu, Lingyan Zhu, Ruhong Zhou, Zhifang Chai, Thomas E. Albrecht-Schmitt, Shuao Wang

**Affiliations:** 1 School for Radiological and Interdisciplinary Sciences (RAD-X), Soochow University and Collaborative Innovation Center of Radiation Medicine of Jiangsu Higher Education Institutions, 199 Ren’ai Road, Suzhou, 215123 China; 2 College of Environmental Science and Engineering, Tianjin Key Laboratory of Pollution Processes and Environmental Criteria, Ministry of Education, Nankai University, 38 Tongyan Road, Tianjin, 300350 China; 3 Computational Biology Center, IBM Thomas J Watson Research Center, Yorktown Heights, NY 10598 USA; 4 Department of Chemistry, Columbia University, New York, NY 10027 USA; 5 Department of Chemistry and Biochemistry, Florida State University, 95 Chieftain Way, Tallahassee, FL 32306 USA

## Abstract

Many environmental pollutants inherently exist in their anionic forms and are therefore highly mobile in natural water systems. Cationic framework materials that can capture those pollutants are highly desirable but scarcely reported. Here we present a mesoporous cationic thorium-based MOF (SCU-8) containing channels with a large inner diameter of 2.2 nm and possessing a high surface area of 1360 m^2^ g^−1^. The anion-exchange properties of SCU-8 were explored with many anions including small oxo anions like ReO_4_^−^ and Cr_2_O_7_^2−^ as well as anionic organic dyes like methyl blue and the persistent organic pollutant, perfluorooctane sulfonate (PFOS). Both fast uptake kinetics and great sorption selectivity toward PFOS are observed. The underlying sorption mechanism was probed using quantum mechanical and molecular dynamics simulations. These computational results reveal that PFOS anions are immobilized in SCU-8 by driving forces including electrostatic interactions, hydrogen bonds, hydrophobic interactions, and van der Waals interactions at different adsorption stages.

## Abstract (web summary)

Cationic metal-organic frameworks provide promising opportunities to capture anionic pollutants, but stable frameworks with sufficiently large pores are lacking. Here the authors present a thorium-based mesoporous, cationic and hydrolytically-stable MOF that can rapidly trap inorganic and organic anionic pollutants.

## Introduction

Cationic metal-organic frameworks (MOFs) represent a small fraction of the MOF family and are much less-explored than neutral and anionic MOFs. Over the past few years, a series of cationic MOFs with transition metals and lanthanides have been designed and synthesized using several different strategies^1^. One method is to react metal cations with neutral N-donor ligands to afford [ML]^+^ frameworks along with unbound or weakly coordinated anions^2–6^. Post-synthetic modification offers a direct route to these materials by converting a neutral framework into a positively charged one^7, 8^. In addition, Bu and colleagues^9^ have reported the systematic synthesis of cationic MOFs based on highly charged metal clusters such as [In_3_O(COO)_6_]^+^ and showed that these materials can be used in anionic dyes separations. Examples of cationic MOFs that are stable in water are sparse in the literature, and even fewer of these exhibit anion-exchange properties^3–6^. If the goal is to remove large anionic species such as organic dyes, pharmaceuticals, or industrial semi-products from water, materials with pore sizes >2 nm are desirable. Hydrolytically stable cationic MOFs with pores of this size have not yet been reported, most likely because preventing the hydrolysis-induced collapse of mesoporous structures is challenging^10, 11^.

Our efforts are focused on utilizing the Th^4+^ cation as a metal center. The Th^4+^ metal node can be used to assemble stable cationic MOFs for several reasons. First, thorium is quite abundant at 11.4 ppm in the earth’s crust^12^, being close to that of lead (13 p.p.m.). Although the predominate isotope Th-232 is slightly radioactive, its specific activity is extremely low at 1.097 × 10^−7^ Ci g^−1^ ^13^. The toxicity of thorium is therefore dominated by its chemical toxicity, which is also demonstrated to be not high and similar to those of Al(III) or Cr(III)^14, 15^. Second, it has already been demonstrated that the incorporation of highly charged metal ions such as Zr(IV) in frameworks often leads to more robust materials^10, 16, 17^ .Third, these tetravalent cations have a tendency to form clusters that lead to expanded frameworks. Th^4+^ mimics Zr^4+^ in this regard^18^, but also offers several features absent with the smaller cation. Among these features are expanded coordination numbers that can reach 12 or higher^19^, and its ability to utilize a variety of frontier orbitals in bonding. This increased covalency may lead to larger bond dissociation energies and hence more resilient materials^20^. These modifications of the nature of coordination and bonding at the metal center allows for the design of both new SBU’s and structural topologies that cannot be replicated in traditional MOFs assembled from transition metals and lanthanides. While Th(IV)’s structural chemistry offers potential advantages, thorium-based MOFs are poorly represented^21–24^, especially when compared with examples containing uranium^25–30^. Albrecht-Schmitt et al. also utilized Th^4+^ cation to construct the first inorganic cationic 3D framework material showing excellent anion-exchange capability for trapping TcO_4_^−^ anion^31, 32^.

During the course of developing new cationic framework materials,^33, 34^ the first mesoporous cationic MOF, [Th_3_(bptc)_3_O(H_2_O)_3.78_]Cl·(C_5_H_14_N_3_Cl)·8H_2_O (SCU-8, H_3_bptc = [1,1′-biphenyl]-3,4′,5-tricarboxylicacid), was discovered. This material is easily prepared as a pure phase via the ionothermal reaction of Th(NO_3_)_4_·H_2_O with H_3_bptc in ionic liquid tetramethylguanidine chloride (C_5_H_14_N_3_Cl) at 140 °C. SCU-8 exhibits superior anion-exchange capability toward a variety of anionic environmental pollutants including small oxo-anions ReO_4_^−^ and Cr_2_O_7_^2−^ as well as organic dyes like methyl blue, and the persistent organic pollutant, perfluorooctane sulfonate (PFOS).

## Results

### Crystal structure depiction

**Fig. 1:**
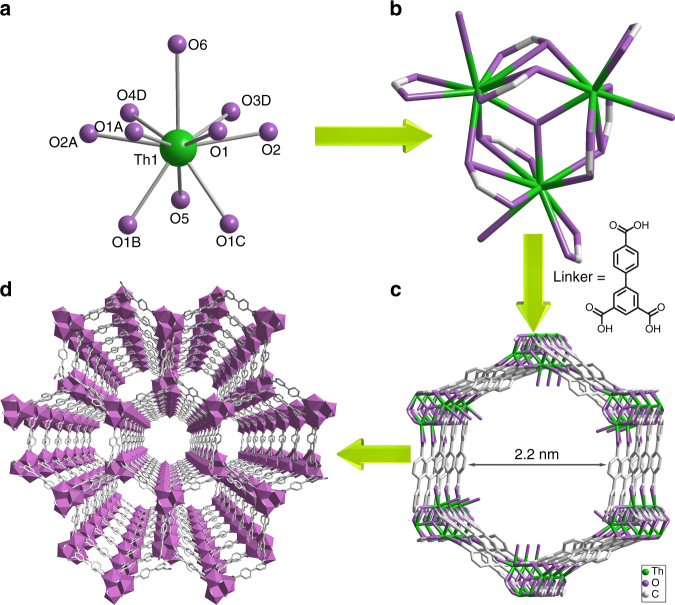
Crystal structure depictions of SCU-8. **a** Coordination geometry of Th^4+^, **b** The cationic cluster of [Th_3_(COO)_9_O(H_2_O)_3.78_]^+^ as the SBU, **c** Hexagonal tubular channels in the structure, **d** A view of the cationic mesoporous framework structure along *c* axis. The carboxylate group (O3, O4), coordinating water (O6) and bptc^3−^ ligand are disordered, and only the major conformations are shown. Atom colors: Th = green, O = purple, C = gray

Single-crystal X-ray diffraction analysis reveals that the compound possesses a 3D open-framework and crystallizes in the hexagonal space group *P*6_3_/*m*. The asymmetric unit contains a half thorium center, a half pbtc^3−^ ligand, one sixth of μ_3_-O atom, and a half coordinating water molecule. The Th^4+^ ion is 10-coordinate and adopts an atypical coordination environment that can be best described as a capped triangular cupola geometry (Fig. 1a)^35^, which has been recently documented for trivalent actinides^36, 37^. Therefore, the Zr^4+^ analog of SCU-8 does not exist because Zr^4+^ is not large enough to be coordinated by ten oxygen atoms^16^. All attempts to prepare the Zr-analog of SCU-8 were unsuccessful. The coordination sphere of thorium contains eight oxygen atoms from five carboxylate groups, one μ_3_-O group, and one coordinating water molecule. Within each thorium coordination sphere, these ten Th-O bonds include six typical thorium carboxyl oxygen bonds ranging from 2.481(9) to 2.563(17) Å, two additional long thorium carboxyl oxygen bonds at 2.8843(4) Å, a thorium μ_3_-O bonds at 2.2684(7) Å and an averaged terminal thorium water oxygen bond at 2.78(7) Å. The relatively long Th-O_w_ distance leads to the initial speculation of a Th-Cl bond instead, which can be however excluded based on the EDS analysis on the Th:Cl molar ratio in the crystal (Supplementary Fig. 1). In addition, such long Th-O_w_ distances are not extremely scarce, which are well-documented in several thorium(IV) compounds^38, 39^. Three thorium atoms are bridged by a μ_3_-O and six μ_2_-O’s from carboxylate groups, forming a cationic [Th_3_(COO)_9_O(H_2_O)_3.78_]^+^ cluster as the secondary building unit of SCU-8 (Fig. 1b). These clusters are further connected by nine chelating/bridging carboxylate groups from the ligands resulting in a 3D cationic network of [Th_3_(COO)_9_O(H_2_O)_3.78_]^+^ with 1D hexagonal tubular channels of 22 × 22 Å (face to face) (Fig. 1c,d). Charge-balancing chloride anions are highly disordered in the channels and cannot be located on the electron density map, but were detected by EDS analysis (Supplementary Fig. 1). Further support of the cationic nature of SCU-8 framework comes from its superior anion-exchange capability (see discussion below) while it is not able to exchange with cations such as Cs^+^, Sr^2+^, and UO_2_^2+^ (Supplementary Fig. 2). In addition, Ionic liquid C_5_H_14_N_3_Cl molecules that were initially used as reaction solvent are present in the channels, as confirmed by combined thermogravimetric and CHN elemental analysis (Supplementary Fig. 3 and Supplementary Table 1). The void volume of SCU-8 was calculated to be 63.8% using PLATON^40^. Therefore, SCU-8 is the most porous thorium compound currently known and one of the most porous actinide compounds in general^21–29^. SCU-8 is also the most porous cationic MOF reported to date^1–9, 41, 42^. Interestingly, it was found that the structure of SCU-8 is closely related to a tetravalent uranium trimesate compound that contains a 10-coordinate U(IV) center, where the terminal oxygen was assigned as a statistical distribution between OH/H_2_O, leading to a neutral framework structure containing much smaller channels. In this compound, no unbound anion was observed in the channels^24, 27^. Overall, combining all the evidences including crystallography, EDS, thermogravimetric and CHN elemental analysis, and ion-exchange studies toward a variety of cations and anions, SCU-8 is demonstrated to be a unique mesoporous cationic MOF compound.

### Surface area and hydrolytic measurement

**Fig. 2:**
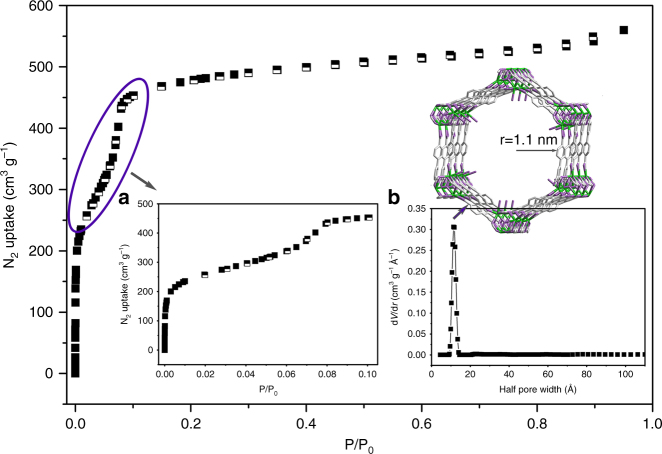
The N_2_ adsorption isotherm for SCU-8 at 77 K. **a** the detail view P/P_0_ between 0–0.1; **b** DFT pores size distribution of SCU-8

The N_2_ sorption isotherm measured at 77 K on an activated SCU-8 sample exhibits type IV behavior (Fig. 2) featuring two plateaus that is typical for mesoporous materials^25, 43^. Calculated using the first plateau, the BET surface area of SCU-8 is 1360 m^2^ g^−1^ (Supplementary Fig. 4), which is the largest reported value among thorium compounds and cationic MOFs. Notably, surface areas were not reported for the majority of cationic MOF compounds^1–9, 42^, likely because of low values originating from relatively small pores and the additional blocking effect induced by the charge-balancing anions in the pores. In addition, many cationic MOFs are not stable under the negative pressure. Among actinide-based MOFs, the surface area of SCU-8 is only smaller than the recently reported uranium-based anionic MOFs NU-1300 (2100 m^2^ g^−1^)^25^ and NU-1301 (4750 m^2^ g^−1^)^29^ and significantly larger than other thorium-based MOFs (TOF-2: 293 m^2^ g;^−1^ Th_6_O_4_(OH)_4_(H_2_O)_6_(bdc)_6_·6DMF·12H_2_O: 730 m^2^ g;^−1^ Th_3_O(btc)_3_(OH)(H_2_O)_2_·2.9DMF·1.5H_2_O: 109 m^2^ g^−1^; [C_9_H_17_N_2_][Th(TPO)Cl_2_]·18H_2_O: 623 m^2^ g^−1^)^21–24^. The pore size of 2.2 nm was calculated based on the Quenched Solid State Functional Theory (QSDFT) and is consistent with the value obtained from the crystal structure (Fig. 2b). The total pore volume of SCU-8 is 0.87 cm^3 ^g^−1^, which is estimated from the N_2_ uptake amount at the normal condition (Fig. 2).

SCU-8 is thermally stable up to 350 °C as shown by the thermogravimetric (Supplementary Fig. 3) and the temperature-dependent powder X-ray diffraction (PXRD) analysis (Supplementary Fig. 5). The hydrolytic stability of SCU-8 was checked by soaking the crystals in aqueous solutions with various pHs for 12 h. Both of PXRD and N_2_ adsorption isotherm measurement results show that SCU-8 partially maintains its crystallinity over the pH range from 4 to 12 (Supplementary Figs 6 and 7). In addition, the PXRD pattern and N_2_ adsorption isotherm were measured on the sample being soaked in aqueous solutions containing various anions, showing mostly retained crystallinity during anion-exchange processes (Supplementary Figs 8 and 9). These results all suggest that the thorium carboxylate oxygen bonds are inert enough to prevent the framework from being completely hydrolyzed. Similar features were also demonstrated in uranyl-based MOF compounds^25, 26^, further highlighting the utility of using actinide ions to build stable MOF materials for applications in nuclear industry.

### Anion-exchange studies

**Fig. 3:**
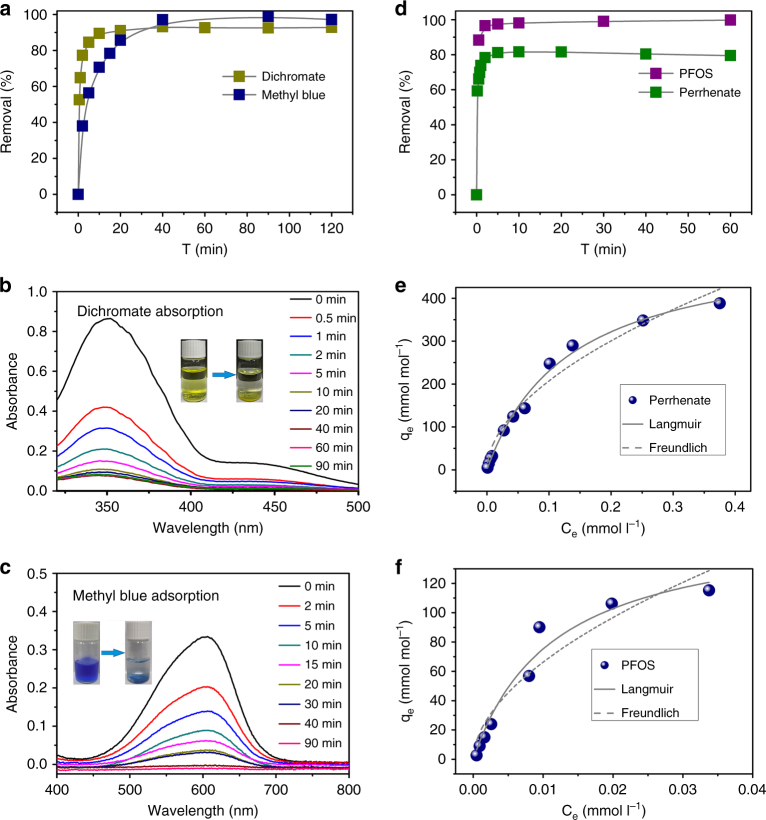
Anion-exchange experiment results. **a** Sorption kinetics of Cr_2_O_7_^2−^ and MB^2−^ on SCU-8; **b** Sorption kinetics of ReO_4_^−^ and PFOS on SCU-8; **c**, **d** UV/Vis absorption spectra of the solutions during the Cr_2_O_7_^2−^ and MB^2−^ uptake processes; **e**, **f** The sorption isotherms of ReO_4_^−^ and PFOS by SCU-8

Anion-exchange experiments were initially performed using anions that are chromophores in the visible region of the electromagnetic spectrum such as Cr_2_O_7_^2−^ and the methyl blue anion (MB^2−^). These exchange experiments were monitored using UV–vis absorption spectroscopy. In the case of the dichromate, max at 353 nm was monitored and showed that more than 89% of Cr_2_O_7_^2−^ was removed from water within 10 min (Fig. 3a). The uptake equilibrium is reached in <30 min with a total removal of 92%, making SCU-8 comparable with microporous cationic MOFs^3, 4, 7, 42^. The success incorporation of guest anions was also confirmed by EDS analyses on the anion-exchanged SCU-8 crystals (Supplementary Figs. 10–13). Similar ion-exchange experiments on MB^2−^ were carried out and showed that SCU-8 is able to quantitatively remove MB^2−^ within 40 min (Fig. 3a). These processes can be also monitored with naked eye as shown in Fig. 3b and c.

The UV–vis data were fitted to a pseudo-second-order kinetics models as provided in Supplementary Table 2. The mesoscale pores of SCU-8 allow it to be the first cationic MOF material that can remove MB^2−^ anions without hydrolytic degradation. These exchange processes are often tested using organic solvents that are not environmentally benign^9, 41^. The removal of large anionic organic dyes from aqueous media is certainly more relevant for environmental remediation.

We also evaluated the uptake capabilities for ReO_4_^−^ as an analog of ^99^TcO_4_^−^, the latter being one of the most problematic species in used nuclear fuel owing to its combined features of high fission yield, long half-life, and high environmental mobility^44^. Crystals of SCU-8 were soaked in a 0.04 mmol l^−1^ ReO_4_^−^ solution with solid-liquid ratio of 2 mmol l^−1^. ICP-MS analysis shows that SCU-8 is able to effectively remove ReO_4_^−^ with a fast kinetics. Approximately 80% of the ReO_4_^−^ anions were removed within 2 min and the sorption equilibrium was reached in less than 10 min (Fig. 3d). The sorption isotherms of ReO_4_^−^ are shown in Fig. 3e and were fit using the Langmuir mode (Supplementary Table 3). This yields a maximum sorption capacity of 534.07 mmol mol^−1^. Therefore, SCU-8 is an attractive candidate for remediating ^99^TcO_4_^−^ contamination in the environment, especially under accidental circumstances that would require a fast response^45^.

Perfluorooctane sulfonate (PFOS) is a global pollutant that was added to the limited/forbidden list at the Stockholm Convention on Persistent Organic Pollutants (POPs) in 2009, primarily due to its combined properties of high chemical stability, bioaccumulation, and toxicity^46^. Environmental Protection Agency (EPA) of United States also established the health advisory levels of 70 ng l^−1^ in drinking water^47^. However, severely polluted water systems that are close to the intensive fluoropolymer facilities can have PFOS concentration of as high as 1.06 mg l^−1^ ^48^, while most other contaminated natural water systems contain PFOS with the concentration ranging from 70 to 1000 ng l^−1^ ^49, 50^. Unlike other well-recognized POPs that are primarily neutral in nature, PFOS is an anionic species and therefore highly soluble and mobile in the environment^51^. The diagonal dimension of PFOS is 1.62 nm defined using a cuboid model. Therefore, SCU-8 should be an ideal candidate for removing PFOS anion from water because PFOS anion can enter the structure of SCU-8 without any orientation limitations.

**Fig. 4:**
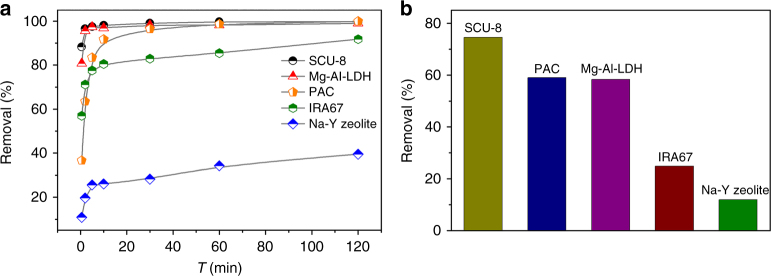
The results of PFOS sorption kinetics and selectivity by various materials. **a** A comparison of PFOS sorption kinetics by SCU-8, Mg-Al-LDHs, IRA67, PAC, and Na-Y zeolite, with the same solid-to-liquid ratio of 0.2 mmol/40 ml and the initial PFOS concentration of 1 mg l^−1^. **b** A comparison of PFOS removal percentages at the equilibrium state in the presence of large excess of multiple competing anions Cl^−^, NO_3_^−^, SO_4_^2−^, and CO_3_^2−^ by SCU-8, Mg-Al-LDHs, IRA67, PAC, and Na-Y zeolite. The concentration of each competing anion is 50 mg l^−1^ and the initial PFOS concentration of 1 mg l^−1^

Impressively, when adding crystals of SCU-8 to a 1 mg l^−1^ PFOS solution, 88 and 96% of the PFOS anions were removed after 30 s and 2 min (and sequentially reached equilibrium as shown in Fig. 3d), respectively, as determined by HPLC-MS/MS. This represents an improvement over minerals, inorganic oxides, anion-exchange resin, and activate carbon in terms of removal kinetics and equilibrium time^51–55^. In order to further support this hypothesis, PFOS sorption kinetics studies for multiple state of art anion sorbent materials including Mg-Al-LDH^56^, state of art anion-exchange resin material IRA67^53^, powdered activated carbon (PAC)^54^, and Na-Y zeolite^55^ were performed under the same conditions (PFOS concentration of 1 mg l^−1^, The identical molar amount of exchangeable anions of each anion sorbent material), in comparison with that of SCU-8. As shown in Supplementary Table 4, the rate constant for the PFOS uptake of SCU-8 is 8.35 mmol mol^−1^ min^−1^, noticeably larger than other existing materials. Figure 4a illustrates that 88.2% of the PFOS anions were removed by SCU-8 after 30 s, lager than other existing materials: 80.7% (Mg-Al-LDH), 36.7% (PAC), 56.9% (IRA67), and 10.9% (Na-Y zeolite). These results highlight the advantage of SCU-8 in terms of sorption rate compared to other materials. Moreover, the PFOS sorption isotherm was fitted using a Langmuir model and gives a maximum sorption capacity of 162.03 mmol mol^−1^ (44.79 mg g^−1^) (Fig. 3f).

Sorption selectivity is critical for practical applications of waste water management and purification, especially for those with high ionic strength. Therefore, we compared PFOS uptake selectivity of SCU-8 with other existing materials through competing ion exchange experiments of PFOS (1 mg l^−1^) in the presence of NO_3_^−^, Cl^−^, CO_3_^2−^, and SO_4_^2−^ (concentrations of each competing anion are in large excess of 50 mg l^−1^). As shown in Fig. 4b, as high as 74% of PFOS can be still removed by SCU-8, which is also noticeably larger than those of Mg-Al-LDH (58%), PAC (59%), IRA67 (25%) and Na-Y zeolite (12%), demonstrating the advantages of SCU-8 in terms of uptake selectivity toward PFOS anion, compared with other materials.

In order to check if SCU-8 is able to remove PFOS at extremely low concentrations that are close to the cases in the real world, another sorption experiment using the solid-to-liquid ratio of 0.51 mmol l^−1^ and PFOS initial concentration of 1 μg l^−1^ was performed. Very promisingly, SCU-8 can rapidly reduce PFOS concentration to 21 ng l^−1^, at least three times lower than the US EPA advisory level of 70 ng l^−1^.

To check the reversibility of anion-exchange process, a variety of desorption solutions including different types of salt solutions (containing NO_3_^−^, Cl^−^, SO_4_^2−^ and/or CO_3_^2−^), acid solutions, basic solutions and methanol solution containing NaCl was used and the best desorption rate is 43.2% using a mixed salt solution containing 1.25% NO_3_^−^, 1.25% Cl^−^, 1.25% SO_4_^2−^, and 1.25% CO_3_^2−^, indicating PFOS anions are partially immobilized in the structure of SCU-8. However, SCU-8 can be still partially regenerated for multiple sorption runs. Although decrease of sorption capacity was observed after the first round, the sorption rate remains almost identical at 44% for sorption/desorption cycles 2, 3, and 4 (Supplementary Fig. 14).

One of the primary concern for the practical application of SCU-8 is the leaching of thorium during anion-exchange process. We therefore investigated the dissolution behavior of SCU-8 in aqueous solution under the same condition of the PFOS sorption kinetics experiment. The dissolution rate of SCU-8 determined by the ICP-analysis of thorium concentration after 5 min, when the sorption equilibrium is reached, is extremely low at ~ 0.003% (Supplementary Fig. 15). This gives a thorium concentration of 9.7 μg l^−1^, which is even much lower than the averaged thorium concentration in the soil^12^, and an activity of 1.06 pCi l^−1^, which is also significantly lower than the maximum α-activity contaminant level of 15 pCi l^−1^ defined by US EPA^57^. The dissolution rate of SCU-8 after 30 min was also determined to be low up to 0.01%. These results further demonstrate that thorium leaching during anion-exchange process may be ignorable, as the result of the high hydrolytic stability of SCU-8 and the large tendency of thorium hydrolysis at neutral pHs^58^.

### The binding pattern and adsorption pathway of PFOS into SCU-8

**Fig. 5:**
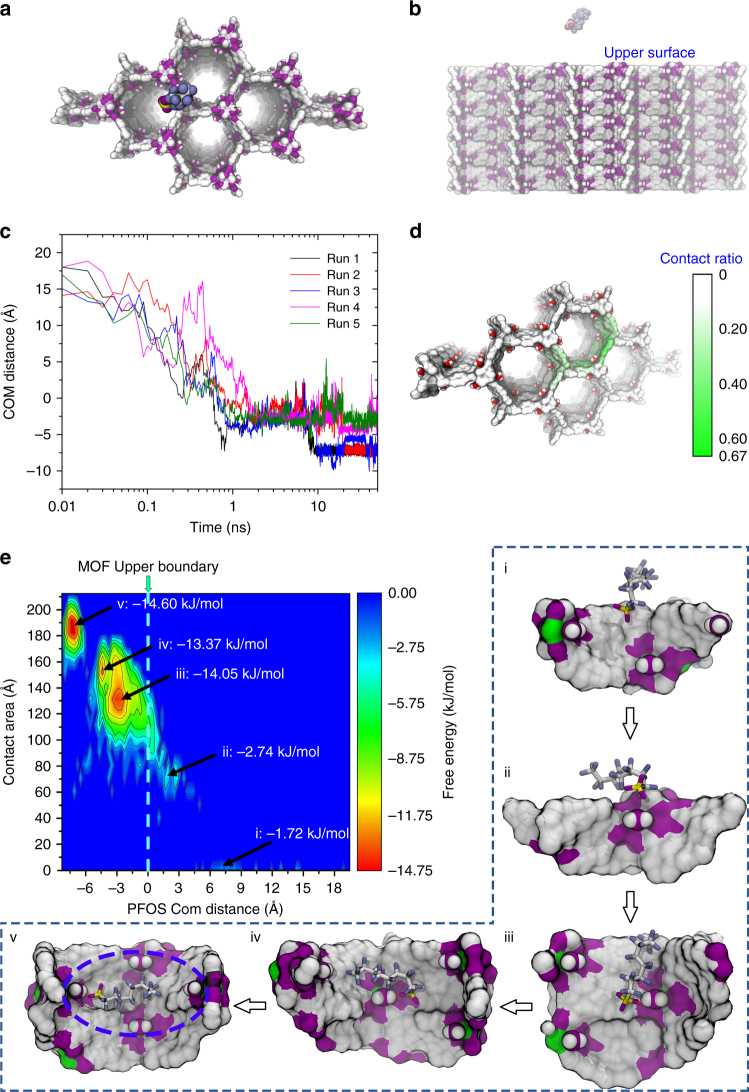
MD simulations on the binding pattern and adsorption pathway of PFOS into SCU-8. The top **a** and side **b** view of simulation system, for clarity only PFOS and SCU-8 are shown; **c** the distance between center of mass (COM) of PFOS and the upper surface of SCU-8 for all five independent runs; **d** the contact ratio between PFOS and SCU-8; **e** the PFOS binding free energy surface (in kJ/mol), which is estimated by W(*A*_cont_, *D*_com_) = −*K*_b_ *T*ln*P*(*A*_cont_, *D*_com_), where *P* is probability of finding the PFOS at position (*A*_cont_, *D*_com_). The color bar for the free energy (in kJ mol^−1^) is given on the right panel figure. Snapshots *i–v* highlighted by the dashed box represent five specific PFOS-SCU-8 binding modes correspond to the five free energy basins

To elucidate the fast sorption processes of PFOS into SCU-8 (see Fig. 5a,b for the initial configuration), as well as to provide insight into the underlying mechanism, all atom molecular dynamics simulations were performed. Within a very short period of time (*t* <1 ns, not including the time for the departure of initial Cl^−^), PFOS was adsorbed into the channels of SCU-8 in all five independent runs, as demonstrated by sharp decrease of the COM (center of mass) distances of PFOS to the upper surface of SCU-8 to below zero (Fig. 5c). Interestingly, the final configurations of five runs can be divided into two different categories: pattern (1) the highly hydrophobic fluorinated alkyl tail of PFOS was fully stacked in a well-defined hydrophobic cavity consisting of benzene rings from the bptc^3−^ ligand while the hydrophilic RSO_3_^−^ group in PFOS formed a hydrogen bond with a coordinating water inside the channel of SCU-8 (run 1 to run 3 in Supplementary Fig. 16); pattern (2) part of the tail of PFOS was still exposed to the bulk water while the RSO_3_^−^ group of PFOS formed a hydrogen bond with a coordinating water inside the channel of SCU-8 (run 4 and run 5 in Supplementary Fig. 16). Further analyses of the contact ratio demonstrate that the well-defined hydrophobic cavity which was encircled by four coordinating water molecules inside the channel of SCU-8 possess the highest contact probability (~ 0.67) with PFOS (Fig. 5d), which suggests that the first binding pattern between PFOS and SCU-8 should be the energetically more favorable binding mode.

To further demonstrate the possible multiple binding modes and adsorption pathway of PFOS to SCU-8, we calculated the binding free energy (potential of mean force, PMF) of PFOS binding to SCU-8. Herein, the binding free energy was computed by W(*A*_cont_, *D*_com_) = −*K*_b_ *T*ln*P*(*A*_cont_, *D*_com_), where *K*_b_ is the Boltzmann constant, *T* is the temperature, *A*_cont_ is the contact area between PFOS and SCU-8, and *D*_com_ is the COM distances of PFOS to the upper surface of SCU-8, and *P*(*A*_cont_, *D*_com_) is the probability of finding PFOS at position (*A*_cont_, *D*_com_). As shown in Fig. 5e, the first relatively shallow free energy basin was located at (7.8 Å, 0.0 Å^2^), with a Δ*G* = −1.72 kJ mol^−1^. In this state, the negatively charged RSO_3_^−^ group of PFOS first attached to SCU-8 (the rest hydrophobic fluorinated alkyl tail of PFOS remains exposed to water) (configuration *i* in Fig. 5), due to the long-range electrostatic interaction between the RSO_3_^−^ group and positively charged SCU-8. At the second free energy basin (2.3 Å, 71 Å^2^), Δ*G* = −2.74 kJ mol^−1^, the RSO_3_^−^ started to throw part of the hydrophobic tail of PFOS into the channel of SCU-8 (configuration *ii* in Fig. 5). At the third free energy basin (−2.9 Å, 131 Å^2^), Δ*G* = −14.05 kJ mol^−1^, the RSO_3_^−^ group formed a firm hydrogen bond with a coordinating water, with a significant portion of the hydrophobic tail of PFOS tightly stacked onto the hydrophobic inner wall of SCU-8 (configuration *iii* in Fig. 5). It is worthwhile to note that the terminal of fluorinated alkyl tail of PFOS was still exposed to water. The binding mode *iii* was very similar to the binding pattern 2. At the fourth free energy basin (−4.3 Å, 115 Å^2^), Δ*G* = −13.37 kJ mol^−1^, different from mode *iii*, the remaining, previously water exposed, fluorinated alkyl tail of PFOS was fully pushed into the channel of SCU-8, due to the strong hydrophobic interaction. During this adjustment process, the fluorinated alkyl tail of PFOS underwent a significant rotation with those tight contacts between PFOS and hydrophobic inner wall of SCU-8 in mode *iii* disassociated and reformed (configuration *iv* in Fig. 5). At the fifth free energy basin (−7.1 Å, 182 Å^2^), Δ*G* = −4.60 kJ mol^−1^, the fluorinated alkyl tail was further pushed into deeper inside the channel of SCU-8, and fully stacked onto a well-defined hydrophobic cavity (configuration *v* in Fig. 5 very similar to binding pattern 1). Therefore, we can deduce that the evolution of binding mode from *iii* (pattern 2) → *iv* → *v* (pattern 1) is the most crucial procedure during its binding process.

### The adsorption dynamics and underlying molecular mechanisms of PFOS into SCU-8

**Fig. 6:**
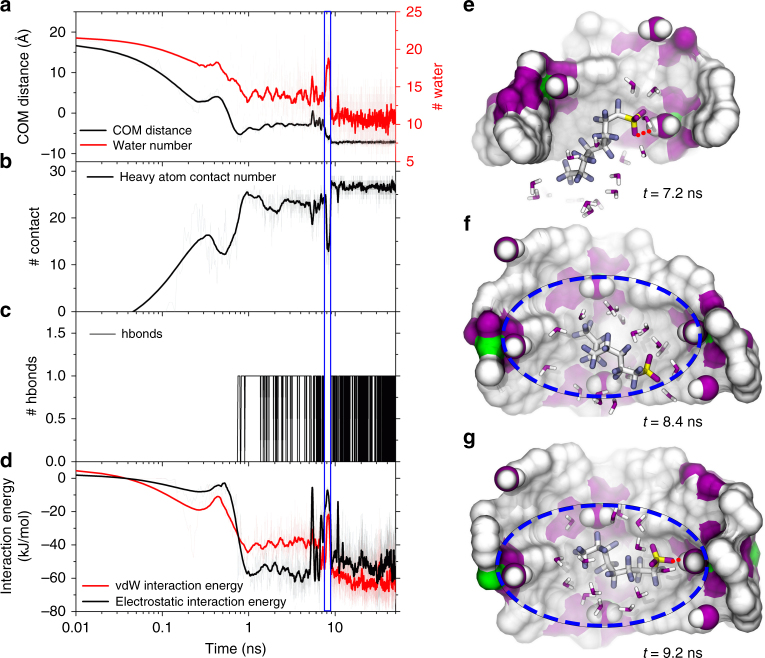
The sorption dynamics of PFOS into SCU-8. A representative trajectory (run 1) to show the adsorption dynamics of PFOS into SCU-8 channel. **a** The distance between center of mass (COM) of PFOS and the upper surface of SCU-8 (left *y* axis, black curve) and the number of water in the first solvation shell of PFOS (right *y* axis, red curve); **b** the heavy atom contact number between PFOS and SCU-8; **c** the number of hydrogen bond formed by RSO_3_^−^ and the coordinating water in the inner wall of SCU-8 channel; **d** time evolution of the electrostatic and van der Waals (vdW) interaction energies between PFOS and SCU-8; **e**–**g** some critical intermediates at *t* = 7.2, 8.4, and 9.2 ns to show the configuration re-adjustment of PFOS binding to SCU-8. The color scheme is the same as Fig. 1. The red dash line stands for the hydrogen bond, and a PFOS specific binding site is highlighted by the blue circle. The coordinating water is shown with vdW balls

To further establish the intrinsic relationship between the sorption behavior and the possible driving forces, a representative trajectory was investigated in detail. Here run 1 was selected as the targeting trajectory because it underwent a transformation from pattern 2 to pattern 1. As shown in Fig. 6a, the COM distance between PFOS and the upper surface of SCU-8 sharply decreased from ~ 18 Å to −5 Å quite quickly (< ~ 0.7 ns). Concomitantly, the heavy atom contact number between PFOS and SCU-8 dramatically increased from 0 to ~ 25 Å (Fig. 6b), demonstrating an ultrafast adsorption of PFOS into the SCU-8. It is noteworthy that accompanying with this adsorption process, water molecules originally residing in between the PFOS and benzene rings of the bptc^3−^ ligand were expelled out of the interfacial region (Fig. 6a red curve). This suggests that the hydrophobic interactions between the fluorinated alkyl tail of PFOS and SCU-8 are one of the key driving forces behind the anion-exchange process. Moreover, a hydrogen bond forms (at *t* = ~0.7 ns) between the RSO_3_^−^ group and the coordinating water molecule pointing toward inside of the channels in SCU-8 (Fig. 6c). To gain further insight into the driving forces for the adsorption processes, time evolution of the electrostatic and vdW interaction energies between PFOS and SCU-8 were further calculated (Fig. 6d). The electrostatic interaction energy decreased from 0 to ~ −60 kJ mol^−1^, while the vdW interaction energy lowered from 0 to ~ −40 kJ mol^−1^, simultaneously. This indicates that the strong electrostatic and vdW interactions collectively drive the early adsorption process, in addition to the hydrophobic interactions.

The hydrogen bond formed (at *t* = ~0.7 ns) between the RSO_3_^−^ group and the coordinating water molecule inside of the channels in SCU-8 (Fig. 6c), anchoring PFOS to the inner wall of channels and providing a prerequisite for the next round of configuration re-adjustment. As shown by a metastable configuration at *t* = 7.2 ns (Fig. 6e), a considerable proportion of the fluorinated alkyl tail of PFOS was still exposed to the bulk water, very similar to binding pattern 2 (run 4 and run 5 in Supplementary Fig. 16). This is thermodynamically unfavorable and a round of configuration re-adjustment (from ~ 7.5 to 9 ns) quickly occurred driven by hydrophobic interactions between the tail of PFOS and the benzene rings of the bptc^3−^ ligand that is strong enough to break the initial hydrogen bond (see a typical configuration at *t* = ~8.4 ns in Fig. 6f). After that, PFOS anions started to transform from binding pattern 2 to pattern 1, with its tail initiated the motion into the deeper space of the channel. Subsequently, strong nanoscale dewetting phenomena^59, 60^ occurs at the interface of the fluorinated alkyl tail of PFOS and benzene rings from bptc^3−^ ligand providing an additional driving force (along with enhanced vdW interactions between PFOS and SCU-8; Fig. 6d red curve; more below) to push the tail fully into the highly hydrophobic cavity. Finally, at *t* = ~9 ns, the RSO_3_^−^ group of PFOS reformed a new hydrogen bond with a different coordinating water in SCU-8 (Fig. 6c,g), thereby firmly anchoring the PFOS anion onto the final binding site till the very end of the simulation.

During this final configuration re-adjustment process, the energy contribution from the vdW interaction (decreases from ~ −40 to ~ −60 kJ mol^−1^, Fig. 6d red curve) is remarkably larger than the electrostatic interaction (stays roughly constant at ~ −60 kJ mol^−1^, Fig. 6d black curve). Overall, our current simulations reveals an interesting microscopic picture for this efficient and fast adsorption of PFOS into SCU-8: the strong hydrophobic interactions between the fluorinated alkyl chain in PFOS, the well-defined hydrogen bond between RSO_3_^−^ group in PFOS and coordinating water in SCU-8, and the vdW interaction together with the strong electrostatic interactions between the SCU-8 cationic framework and the PFOS anions all play some important roles at different stages, which collectively drive PFOS anions into the interior channels of SCU-8, followed by a firm immobilization. This is quite significant given that typical PFOS adsorbent materials reported up to date would involve only one or two of these driving forces^51^, and offers clues on why SCU-8 exhibits such advantages in term of sorption kinetics and efficiency over other reported sorbent materials. Moreover, in order to further validate the robustness of the force field parameters and treatment of the long-range electrostatic interactions with PME method^61^, in our current study, we have performed an additional set of simulations with the larger sized SCU-8 cluster (a 8 × 4 × 5 supercell, containing 15,520 atoms, see Supplementary Fig. 17a, b, which is about twice the size of that of the former smaller cluster, a 3 × 3 × 5 supercell, containing 8730 atoms, Fig. 5a,b). We found that the timescale of PFOS uptake (Supplementary Fig. 17c) and the binding pattern (Supplementary Fig. 17d–f) are all comparable to the above results, i.e., independent of the cluster size, indicating a reasonable choice of the simulation models and methods in our current study.

## Discussion

In summary, SCU-8 is a rare example of a hydrolytically stable, mesoporous 3D cationic MOF with superb anion-exchange capabilities for a variety of anions including small oxo-anions and relatively large organic anions such as MB^2−^ and PFOS. The cationic framework structure of SCU-8 is based on a unique cationic SBU of [Th_3_(COO)_9_O(H_2_O)_3_]^+^ containing atypical 10-coordinate Th^4+^ ions, further highlighting the uniqueness of actinides in building new MOF structure that cannot be mimicked by transition metals and lanthanides. In addition, this combined approach of both experimental and computational techniques reveals a new mechanism for SCU-8 in its adsorption of PFOS that involves multiple driving forces at different adsorption stages, making SCU-8 one of the best scavengers of the PFOS anion. This work sheds light on the design of tailored MOF structures with desired charge, proper pore size/shape, hydrophilic/hydrophobic component arrangement and functionalities for the efficient removal of targeted environmental pollutants.

## Methods

### Materials

Although the chemotoxicity and radiotoxicity of thorium are in general considered to be low, the standard procedure for handling radioactive materials should be followed. Mg-Al-LDH was synthesized according to the literature^56^ and the formula is Mg_2_Al(OH)_6_NO_3_·4H_2_O. The Amberlite IRA-67 free base (complete exchange capacity of 1.6 eq l^−1^), power activated carbon (PAC 400 mesh) and Na-Y zeolite (molar ratio of Na:Si = 2:5.1) were purchased from Alfa Aesar. All other reagents and solvents were used as received from commercial suppliers (Adamas or Aladdin) without further purification.

### Synthesis of SCU-8

H_3_bptc (28.63 mg, 0.1 mmol), Th(NO_3_)_6_·6H_2_O (58.8 mg, 0.1 mmol) and ionic liquid of tetramethylguanidine chloride (227.4 mg, 1.5 mmol) were placed in a 23 ml PTFE-lined autoclave. Then 3 drops of concentrated nitric acid were added to the mixture. The mixture was then sealed and heated at 140 °C for 2 days. The reaction system was cooled to 30 °C in 24 h. After filtration and washed with excess of DMA and ethanol, colorless block crystals were collected in a yield of ca. 60 % (based on thorium) as a pure phase (see PXRD data in Supplementary Fig. 18). Zr(IV) analog reaction was also investigated but failed to yield any crystalline phases.

### Crystallographic analysis

Data collection was accomplished on a Bruker D8-Venture diffractometer with a Turbo X-ray Source (Mo–Kα radiation, *λ* = 0.71073 Å) adopting the direct-drive rotating anode technique and a CMOS detector at 173 K. The data collection was carried out by using the program APEX3 and processed using SAINT routine in APEX3. The structure of SCU-8 was solved by direct methods and refined by the full-matrix least squares on *F* ^2^ using the SHELXTL-2014 program. All non-hydrogen atoms were refined with anisotropic displacement parameters. Hydrogen atoms attached to carbon atoms were placed in geometrically idealized positions. Merohedral twinning with the twin law of (1 1 0 0 −1 0 0 0 −1) was observed in the structure solution under the space group of *P*6_3_/*m*. ISOR restraint was applied to light atoms C and O that are close to Th center. Crystallographic data of SCU-8 is summarized in Supplementary Table 5. Selected bond lengths for SCU-8 are listed in Supplementary Table 6. In this compound, the coordinated water bound to Th center was assigned as a statistical distribution over two positions. The charge-balancing chloride anion and ionic liquids are highly disordered and impossible to refine using conventional discrete-atom models. To resolve this issue, the contribution of solvent-electron density of 1819 electrons per unit cell was removed using the SQUEEZE routine in PLATON, thereby producing a set of solvent-free diffraction intensities. The final formula of SCU-8 was calculated in combination with those of elemental analyses and TGA. The solvent content determined by TGA is significantly lower than that determined by crystallography, because of the loss of solvent induced by heating operation before the analysis.

### Physical property measurements

The elemental analyses (C, H, and N) were carried with a Vario EL CHNOS elemental analyzer. The powder X-ray diffraction (PXRD) patterns were collected from 3° to 50° with a step of 0.02° and 0.5 s on a Bruker D8 Advance diffractometer with Cu–Kα radiation (*λ* = 1.54056 Å) and a Lynxeye one-Dimensional detector. Thermalgravimetric analysis (TGA) was performed on a NETZSCH STA 449F3 instrument in 30–900 °C under a nitrogen flow at a rate of 10 °C min^−1^. TGA sample was prepared by heating SCU-8 solid in an oven at 60 °C for 12 h before analysis. A Quantachrome Autosorb Gas Sorption analyzer IQ2 was used to perform N_2_ adsorption measurements. Scanning electron microscopy/energy-dispersive spectroscopy (SEM/EDS) images and data were collected using FEI Quanta 200 FEG. The concentration analysis of Re was completed using a Thermo Finnigan high resolution magnetic sector Element 2 ICP-MS instrument. HPLC-MS/MS analysis of PFOS concentration in solution was conducted using a Waters Xevo TQ_S instrument. The UV–vis spectroscopy was collected by using a Thermo Scientific GENESYS 10 s UV–vis Spectrophotometer. Single-crystal X-ray analysis was accomplished on a Bruker D8-Venture diffractometer with a Turbo X-ray Source (Mo–Kα radiation, λ = 0.71073 Å) adopting the direct-drive rotating anode technique and a CMOS detector at 273 K.

### Surface area measurements

Gas adsorption measurements were carried at 77 K with a liquid nitrogen bath, the detecting pressures range from 0 to 760 Torr. Before testing the sample was added to the methanol solution of LiNO_3_ and the solution was replaced with fresh one every 6 h for 2 days. The sample was further treated with methanol to remove excess LiNO_3_. After spilling the methanol, the sample was activated using the outgas function of Gas Sorption analyzer IQ2 for 10 h at 80 °C. The linear P/P_0_ range (0.01–0.05) in the first plateau was selected to calculate surface area. The distribution of pore size was determined by using the model N_2_ at 77 K on carbon (cylinder pores, QSDFT adsorption branch model) of the ASiQwin software.

### Stability investigations

Hydrolytic stability measurements for SCU-8 were carried out by soaking 0.02 mmol (40 mg) SCU-8 samples in 40 ml aqueous solutions with various pHs (2–12) for 12 h. Thermal-stability measurements were completed by heating 0.03 mmol (60 mg) SCU-8 samples to different temperature (100 °C~700 °C) using a muffle furnace in air.

### Anion-exchange experiments

Dichromate anion uptake by SCU-8 was studied by soaking 0.03 mmol (60 mg) of SCU-8 in 15 ml of 0.3 mmol l ^−1^ Cr_2_O_7_^2−^ aqueous solution. Similarly, the MB^2−^/ReO_4_^−^ sorption measurements were carried by adding 0.03 mmol (60 mg) of SCU-8 in 15 ml aqueous solutions containing 0.02 mmol l^−1^ MB^2−^ or 0.04 mmol l^−1^ ReO_4_^−^. The concentration of Cr_2_O_7_^2−^ and MB^2−^ were monitored by UV–vis Spectrophotometer. The analysis of Re concentration was completed using a ICP-MS instrument.

The sorption kinetics of PFOS anion on SCU-8 were studied by steeping 0.1 mmol (200 mg) solid sample in 40 ml 1 mg l^−1^ PFOS solutions. The sorption kinetics of PFOS by various existing materials including Mg-Al-LDH, IRA67, PAC and Na-Y zeolite were measured under the same conditions (The identical molar amount of exchangeable anions of each anion sorbent material) with that of SCU-8 for comparison. All sorption kinetics conform to the pseudo-second-order model (*t/q*_t_ = 1/*h* + *t/q*_e_, where *q*_t_, *q*_e_ represent the amounts of adsorbate at certain time *t* or at equilibrium time, *h* is the initial adsorption rate, *h* = *kq*_e_^2^ and *k* is the rate constant), the obtained parameters are shown in Supplementary Table 2 and Table 4.

The sorption isotherms of ReO_4_^−^/PFOS in SCU-8 were determined by adding 0.0077 mmol (15 mg) solid samples into 15 ml solutions with various anion concentrations. The Langmuir and Freundlich models were used to interpret the experimental data and Supplementary Table 3 lists the resulting best-fitted model parameters. In addition, the time dependent leaching of thorium ions into the solution was monitored by ICP-MS.

The influence of the competing ions was studied using the mixed anion solutions containing NO_3_^−^, Cl^−^, CO_3_^2−^, and SO_4_^2−^ (concentrations of each competing anion are in large excess of 50 mg l^−1^). 0.1 mmol (200 mg) of SCU-8 was added to 40 ml mixed solution and stirring for 2 h, then the final solution was filtered and the concentration of PFOS was determined by HPLC-MS/MS. The selectivity experiments of other existing materials including Mg-Al-LDH, IRA67, PAC and Na-Y zeolite were carried out following the same condition and procedure (The identical molar amount of exchangeable anions of each anion sorbent material). The sorption experiment at low concentration was completed by immersing 0.01 mmol (20 mg) solid samples to 20 ml 1 μg l^−1^ PFOS solution. After 2 h, the concentration of PFOS in final solution was analyzed by HPLC-MS/MS.

The desorption experiment was carried by dispersing 0.02 mmol (40 mg) SCU-8 into 40 ml 1 mg l^−1^ PFOS solution then stirred for approximately 2 h at room temperature to prepare the PFOS-exchanged sample. After separation by centrifugation and dried in the air, the PFOS-exchanged sample was dispersed into 40 ml solution containing 1.25% NO_3_^−^, 1.25% Cl^−^, 1.25% SO_4_^2−^ and 1.25% CO_3_^2−^, for another 2 h to desorb the PFOS anion. The sampling solutions were filtered with a 0.22 μm nylon membrane filter and the concentrations of PFOS were measured by HPLC-MS/MS. The regenerated sample was again used for PFOS uptake under the same condition described above and four sorption/desorption cycles were performed and analyzed.

### Molecular dynamic simulations

First-principle calculations based on density functional theory (DFT) were performed using Gaussian 09 program to derive the atomic electrostatic potential fit charges (ESP fit charges)^62^. The smallest unrepeatable unit (Supplementary Fig. 19a) was selected as the computational model, which was extracted from the experimentally obtained crystal structure of SCU-8. Single-point energy calculation was performed for the model at B3LYP/RECP~ 6–31 G* level. The Becke three parameters hybrid exchange-correlation functional (B3LYP) was employed^63, 64^. The standard Gaussian-type basis sets 6–31 G* was used for all light elements, including C, O and H atoms^65^. The relativistic effective core potential (RECP) ECP60MWB^66^ and its corresponding (14s13p10d8f6g)/[10s9p5d4f3g] valence basis set which could introduce the scalar relativistic effects, were used for Th atoms^67^. On the basis of the ground state electron density, the atomic charges fit to the electrostatic potential at points selected according to the CHelp scheme (using the Chirlian-Francl model) were deduced^68^. During fitting atomic charges, ten concentric layers of points were used for each atom and more than 7000 points in total were used for fitting atomic charges to ensure the accuracy. The resulting atomic charges were then used for the subsequent classical MD simulations.

The initial configuration of SCU-8 cluster used in the MD simulations was derived from a 3 × 3 × 5 supercell (containing 8730 atoms) obtained from the crystal structure (Supplementary Fig. 19b). Partial charges for atoms were obtained from DFT calculations on the asymmetric unit of SCU-8. The Lennard–Jones (L–J) parameters for all atoms were extracted from OPLS-AA force field^69^. The Lorentz–Berthelot combining rules were adopted to calculate cross L–J interaction parameters. Many molecular simulation studies have shown good agreements with experimental results^70–72^. The reasonable accuracy of the OPLS-AA force field was also validated in describing the microcosmic pictures of adsorption and diffusion in various MOFs. Initially, SCU-8 was solvated into a rectangular water boxes with the size of (124 Å × 87 Å × 120 Å), then 180 Cl^−^ were added to neutralize the system. After two 1 ns NVT equilibrium, the PFOS was then introduced into water box, with a center of mass distance between PFOS and the upper surface of SCU-8 at least 1.5 nm. Meanwhile, to neutralize the simulation system again, a Cl^−^ were removed to balance the charges of PFOS. Consequently, the system contains a SCU-8 framework, a PFOS anion and 179 Cl^−^. The OPLS-AA force field was adopted for PFOS^73^. The resulted simulation system containing 38,987 water molecules. This fully solvated complex was then simulated with MD simulations, which are widely adopted in the studies of biomolecules^59, 74^ as well as nanomaterials^60^. Moreover, in order to validate the robustness of parameters and treatment of the long-range electrostatic interactions with particle-mesh Ewald (PME) method^61^, we have performed an additional set of simulation with the larger sized SCU-8 cluster (a 8 × 4 × 5 supercell, containing 15,520 atoms, Supplementary Fig. 18a and b). The system setup protocols of the larger SCU-8 cluster follow that of the former smaller one. The final simulation box, with a size of (160 Å × 120 Å × 120 Å), contains a PFOS anion, 76,298 water molecules, and 319 Cl^−^.

The software package GROMACS-5.0.2 was used for all MD simulations^75^. The VMD software^76^ was used for trajectory visualization and analysis. The TIP3P water model^77^ was used to represent water molecules. During the simulation, temperature was maintained at 300 K by using *v*-rescale thermostat^78^. Periodic boundary conditions were applied in all three directions. All simulated SCU-8 clusters were frozen throughout the simulation process. Long-range coulomb interactions were computed using PME method^61^, while the van der Waals (vdW) interactions were treated with a simple cutoff scheme (12 Å). The LINCS algorithm was used to maintain all solute bonds at their equilibrium values^79^, and the SETTLE algorithm was used to constrain water geometry^80^. During the production runs, five and three independent of 50 ns repeats were performed for the small and large sized SCU-8 clusters, respectively. A time step of 2.0 fs was used, and data were collected in every 5 ps. All production runs were carried out in the NVT ensembles. The total accumulated simulation time was larger than 400 ns.

### Data availability

The X-ray crystallographic coordinates for structure reported in this study is provided as cif file in Supplementary Data 1 and have been deposited at the Cambridge Crystallographic Data Centre (CCDC), under deposition numbers 1569612. This data can be obtained free of charge from The Cambridge Crystallographic Data Centre via HYPERLINK “http://www.ccdc.cam.ac.uk/data_request/cif”.

## Electronic supplementary material

Supplementary Information

Description of Additional Supplementary Files

Supplementary Data 1
