# Supplementary material for: A mesoporous cationic thorium-organic framework that rapidly traps anionic persistent organic pollutants
Source: Nat Commun. 2017 Nov 7;8:1354. doi: 10.1038/s41467-017-01208-w (PMC5677036; doi:10.1038/s41467-017-01208-w)
Supplement: Supplementary file 1 — Supplementary Information [file 41467_2017_1208_MOESM1_ESM.pdf]

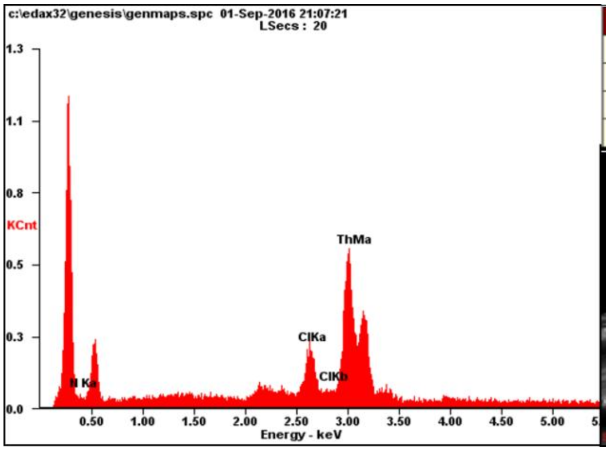

Element	Wt%	At%
NK	07.90	48.25
ClK	08.69	20.99
ThM	83.41	30.77
Matrix	Correction	ZAF

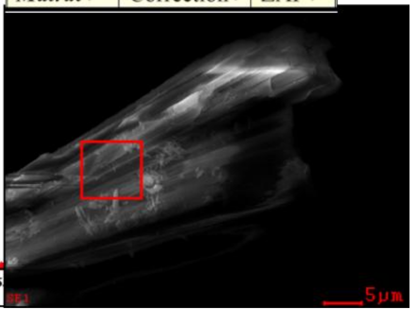

Supplementary Figure 1. The EDS analysis results for SCU-8.

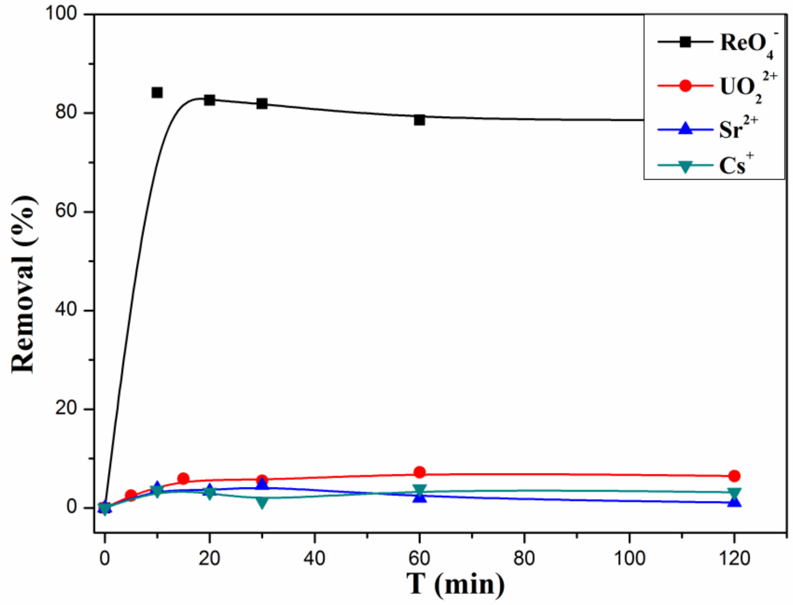

Supplementary Figure 2. Removal kinetics of ReO_4^- (1 mg l^{-1}), Sr^{2+} (1 mg l^{-1}), Cs^+ (1 mg l^{-1}), and UO_2^{2+} (5 mg l^{-1}) by SCU-8 (solid-to-liquid ratio: 1 mg ml^{-1}).

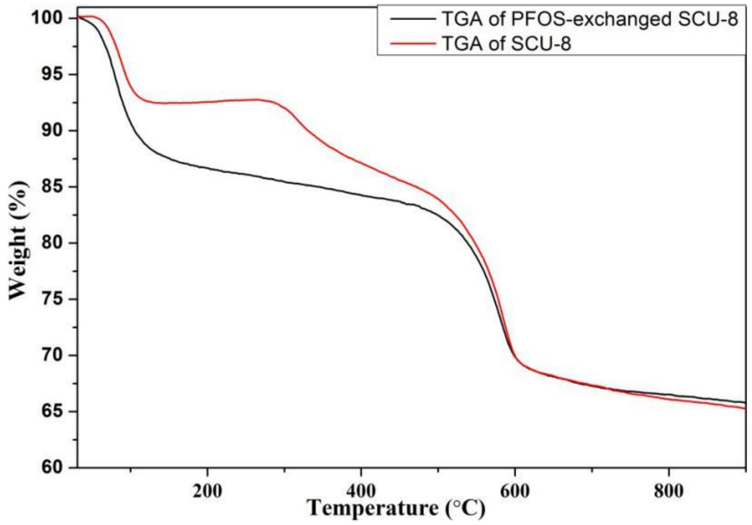

Supplementary Figure 3. The TGA curve for PFOS-exchanged SCU-8 and SCU-8. For SCU-8, three weight loss steps are observed in SCU-8: the first loss 7.3% occurs before 120 ℃, which is attributed to the loss of free waters (cal. 7.39%); the second loss 7.4% between 277 ℃ and 467 ℃ is assigned to the decompose of the ionic liquid (7.79%), and the third weight loss represents the decompose of the bptc^{3-} ligand, leading to the framework collapse. For PFOS incorporated SCU-8, three weight loss steps are also observed in PFOS-exchanged SCU-8: The first loss occurs before 120 ℃, which is attributed to the loss of free waters; the second loss between 120 ℃ and 450 ℃ is assigned to the decompose of the ionic liquid and PFOS anion, and the third weight loss represents the decompose of the bptc^{3-} ligand, leading to the framework collapse.

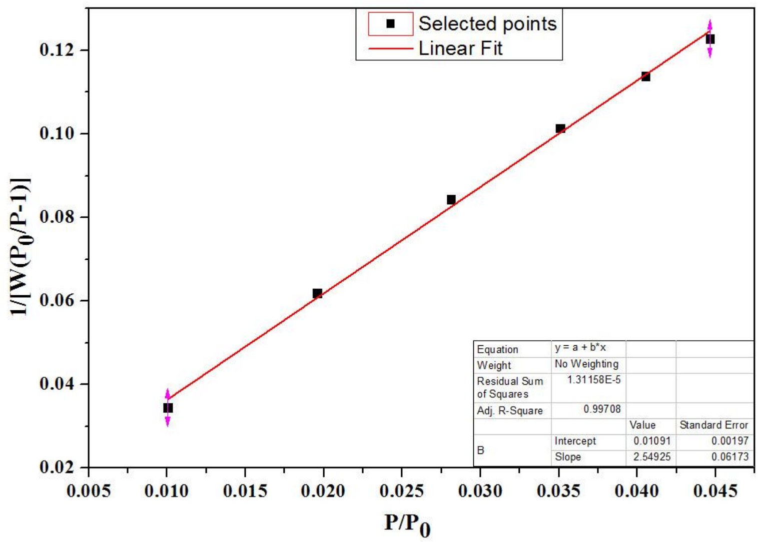

Supplementary Figure 4. BET surface area calculation for SCU-8.

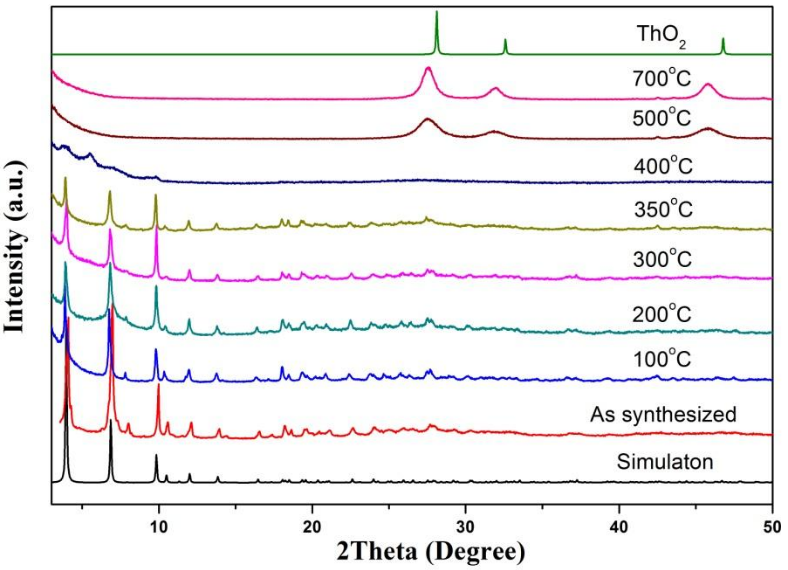

Supplementary Figure 5. Temperature dependent PXRD measurement results of SCU-8.

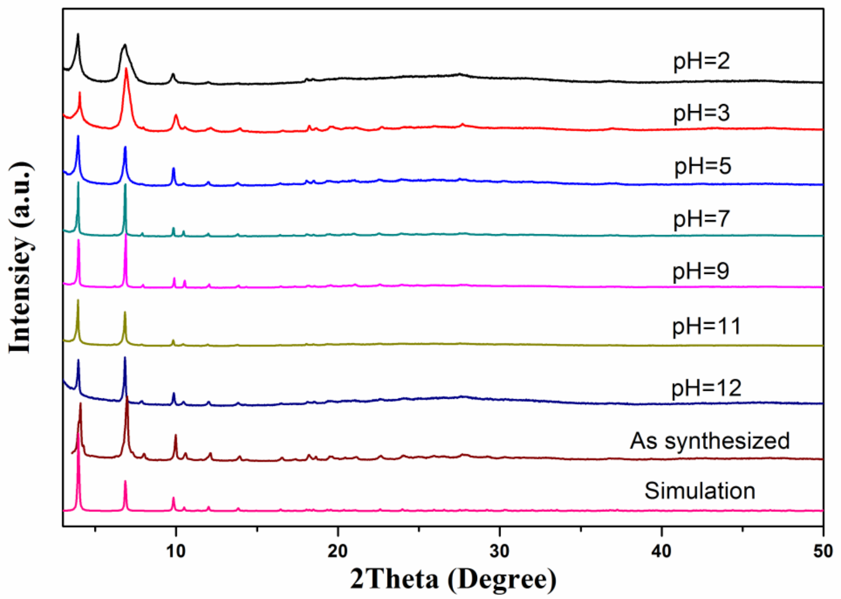

Supplementary Figure 6. PXRD patterns of SCU-8 after soaking in aqueous solutions with different pH values ranging from 2 to 12 for 12 h.

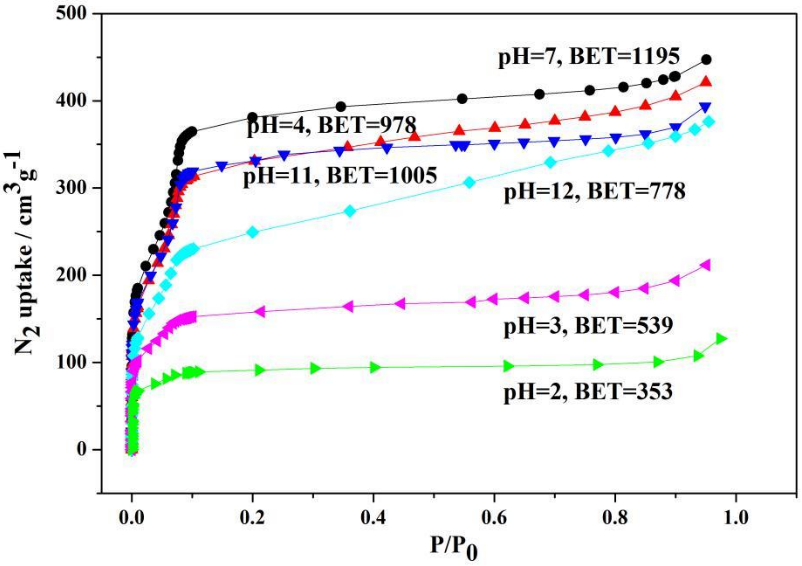

Supplementary Figure 7. The N_2 adsorption isotherm of SCU-8 after soaking in aqueous solutions with different pH values ranging from 2 to 12 for 12 h.

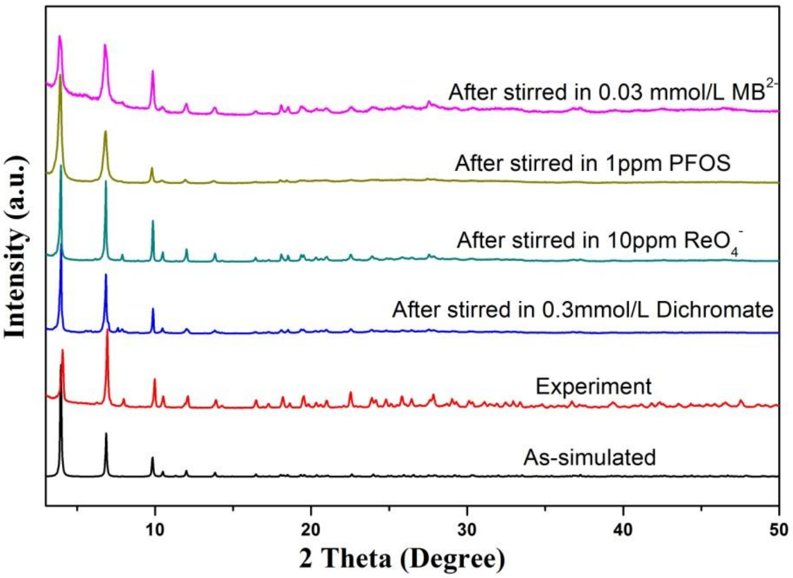

Supplementary Figure 8. The PXRD for SCU-8 after soaking in different solutions containing targeted anions.

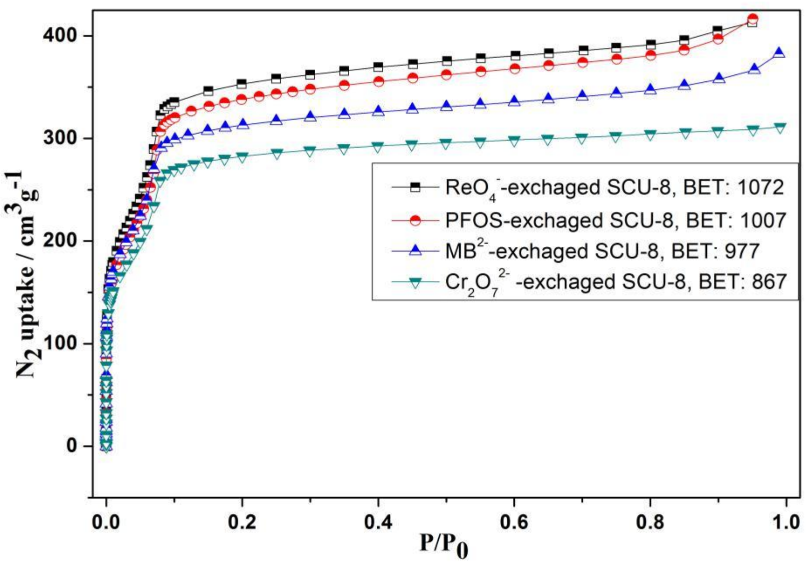

Supplementary Figure 9. The N_2 adsorption isotherm of SCU-8 after soaking in different solutions containing targeted anions.

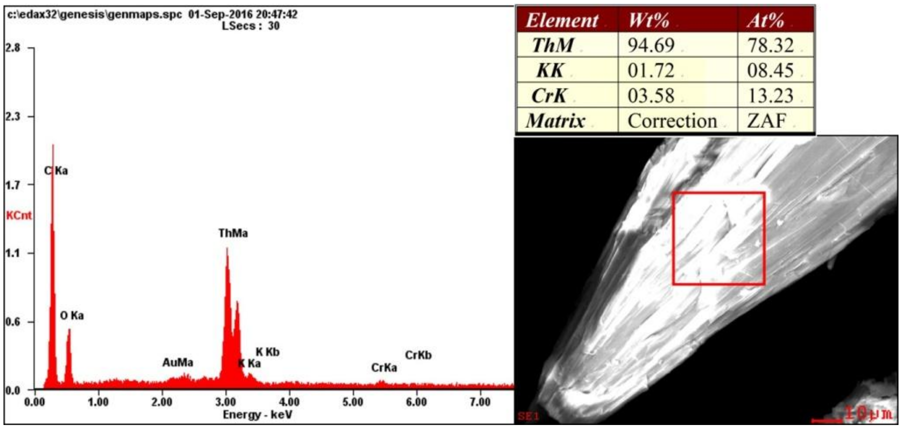

Element	Wt%	At%
ThM	94.69	78.32
KK	01.72	08.45
CrK	03.58	13.23
Matrix	Correction	ZAF

Supplementary Figure 10. The EDS analysis results for SCU-8 after soaking in dichromate solution.

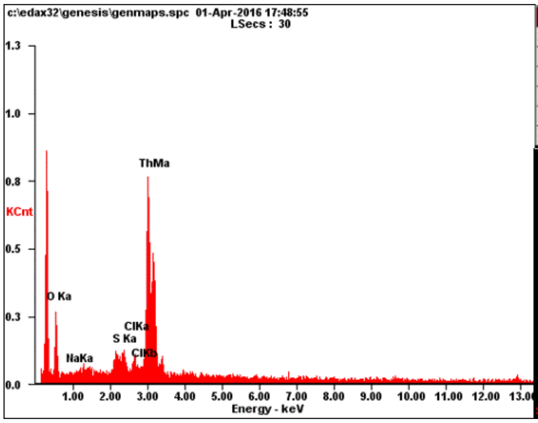

Element	Wt%	At%
OK	19.46	71.92
NaK	00.16	00.41
SK	02.59	04.78
ClK	02.17	03.62
ThM	75.63	19.27
Matrix	Correction	ZAF

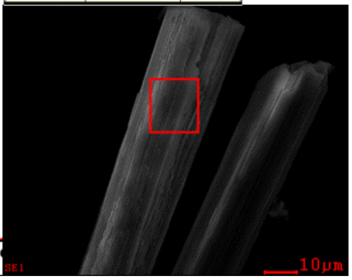

Supplementary Figure 11. The EDS analysis results for SCU-8 after soaking in MB^{2-} solution.

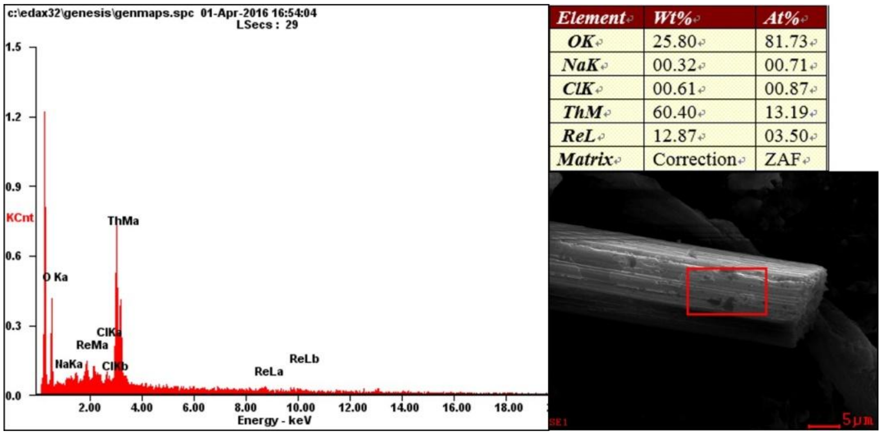

Element	Wt%	At%
OK	25.80	81.73
NaK	00.32	00.71
ClK	00.61	00.87
ThM	60.40	13.19
ReL	12.87	03.50
Matrix	Correction	ZAF

Supplementary Figure 12. The EDS analysis results for SCU-8 after soaking in ReO_4^- solution.

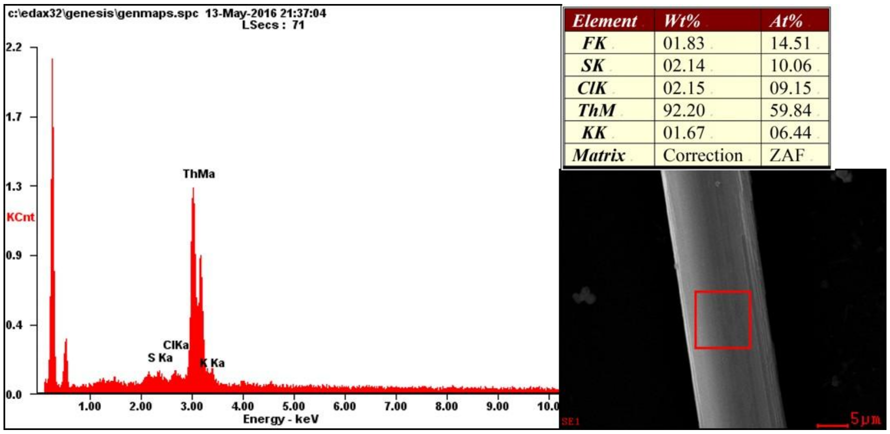

Element	*Wt%*	*At%*
FK	01.83	14.51
SK	02.14	10.06
ClK	02.15	09.15
ThM	92.20	59.84
KK	01.67	06.44
Matrix	Correction	ZAF

Supplementary Figure 13. The EDS analysis results for SCU-8 after soaking in PFOS solution.

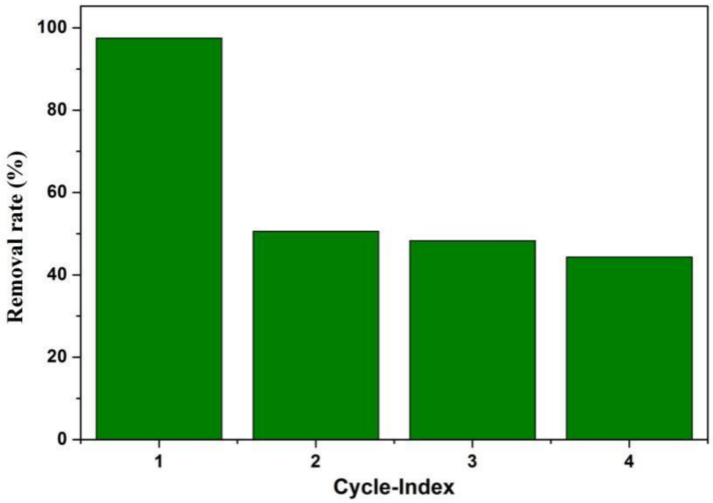

Supplementary Figure 14. The PFOS removal percentages of SCU-8 for 4 sorption/desorption cycles. The solid-to-liquid ratio is 0.5 mmol l^{-1} and the initial PFOS concentration is 1 mg l^{-1}.

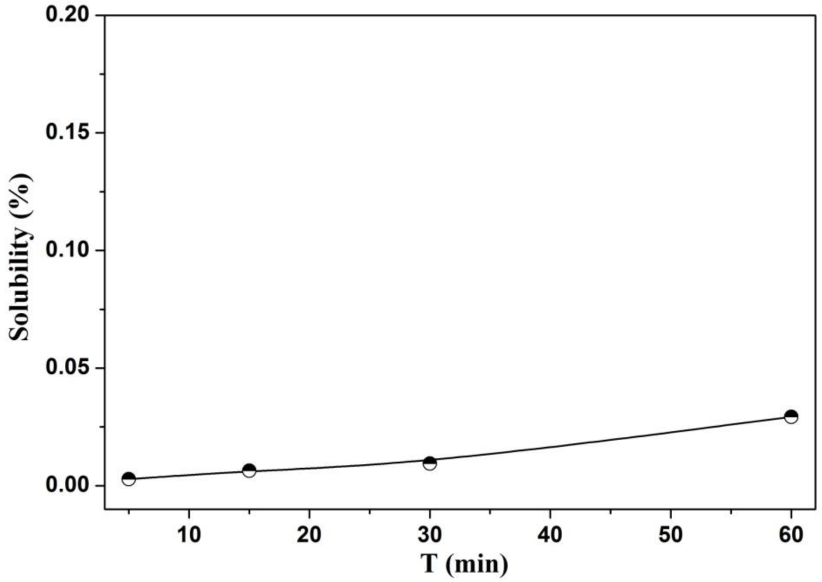

Supplementary Figure 15. The solubility of Th (aq) for dissolution experiments using the 15 mg SCU-8 in 15 ml PFOS solution.

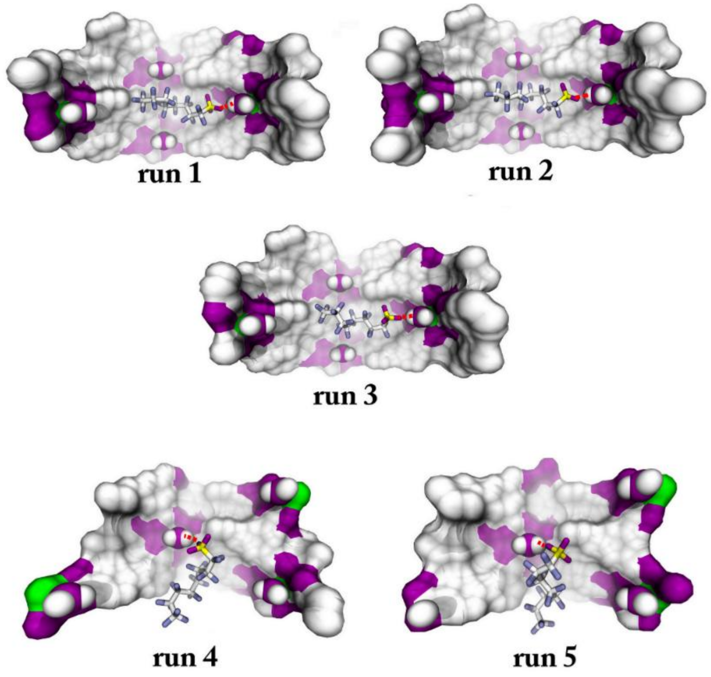

Supplementary Figure 16. The final snapshots for all five runs.

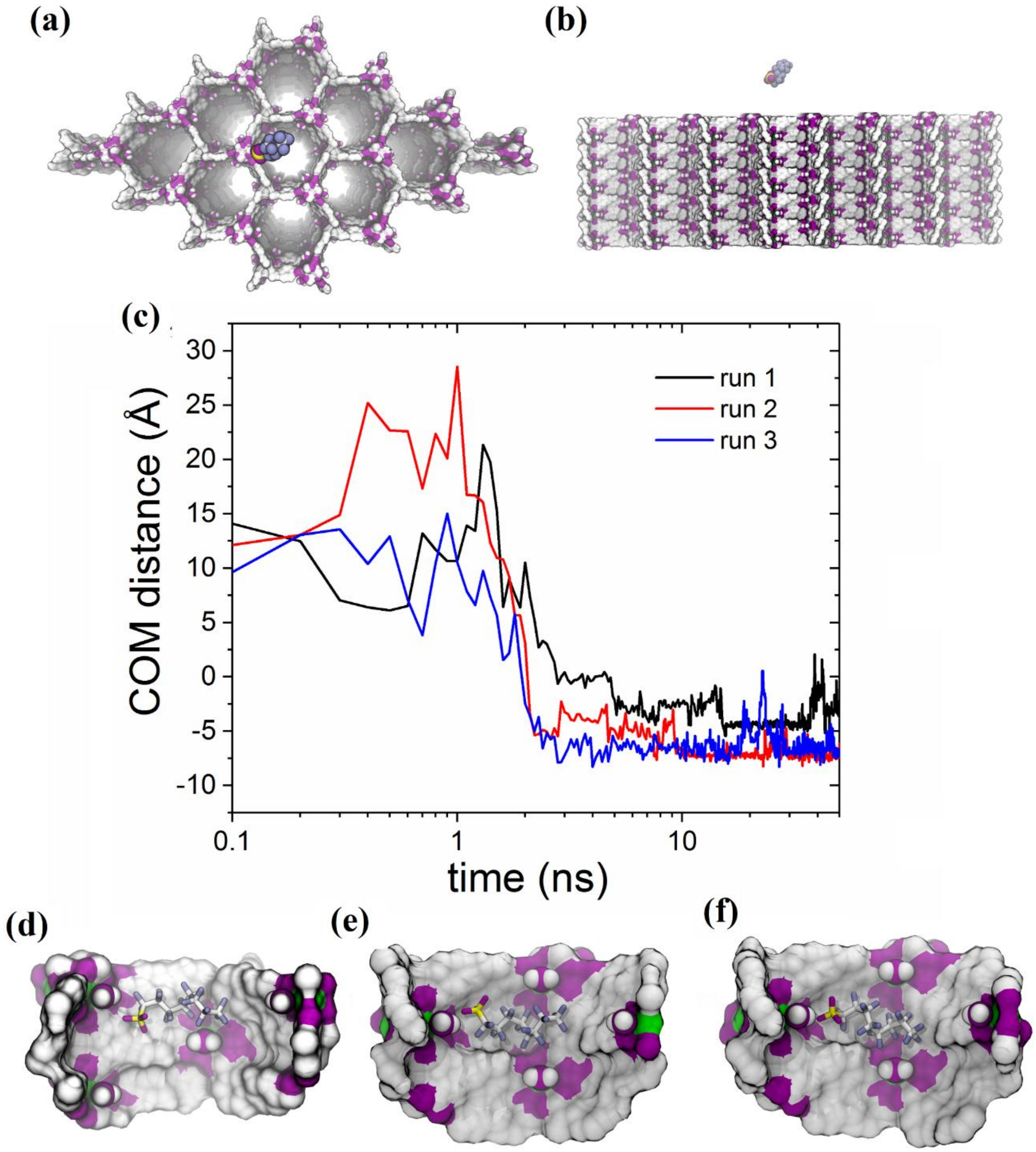

Supplementary Figure 17. The top (a) and side (b) view of simulation system of larger SCU-8 material, for clarity only PFOS and SCU-8 are shown; (c) the COM distance between PFOS and the upper surface of SCU-8 for all three independent runs; the final snapshots for all three runs.

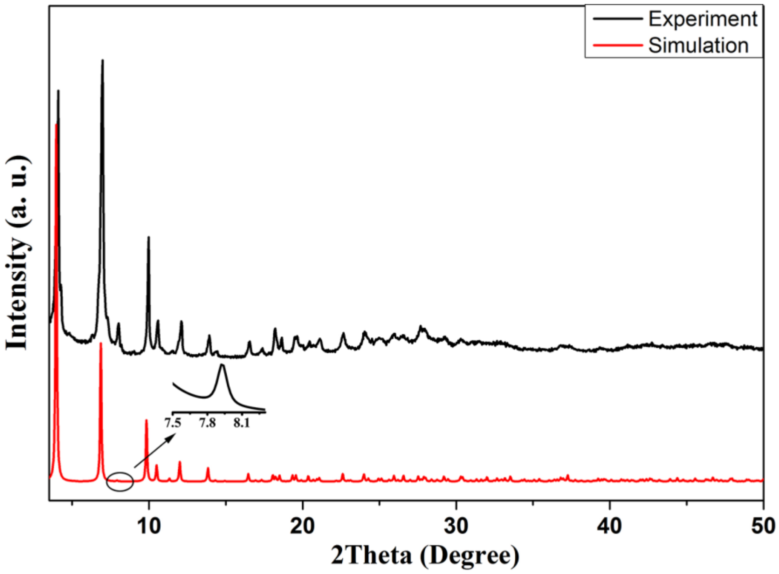

Supplementary Figure 18. The simulated and synthesized powder X-ray diffraction (PXRD) patterns for SCU-8. The inset shows that the peak at ca. 8 °is not an indication of an impurity, which is in fact included in the simulated pattern.

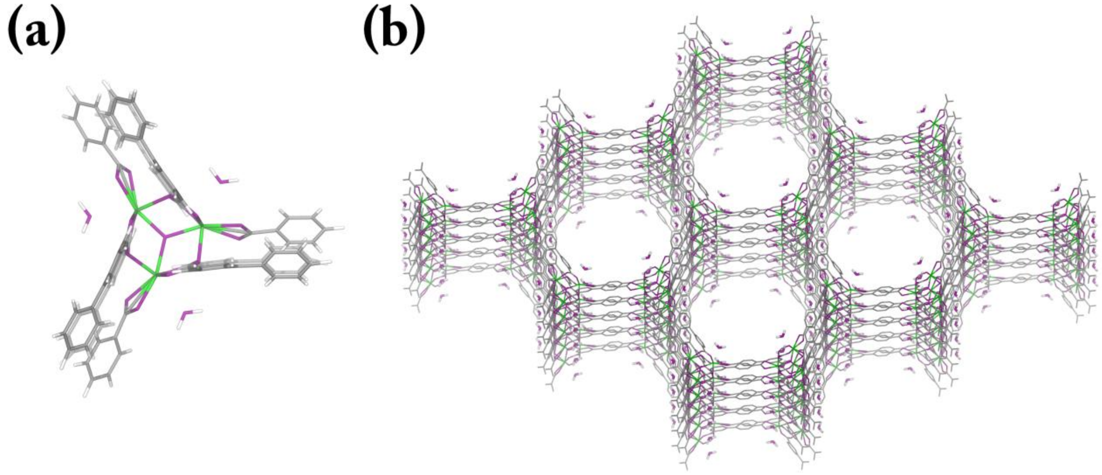

Supplementary Figure 19. (a) The smallest unrepeatable unite extracted from the SCU-8 unit cell which was chosen as the DFT computational model (the chemical formula is $O^{2-}(Th^{4+})_3(C_{12}H_9COO^-)_6(C_6H_5COO^-)_3(H_2O)_3$). The green, purple, gray and white balls indicate the Th, O, C and H atoms. The dangling carbon atoms of the fault surfaces were terminated by H atoms. (b) Schematic diagram of the MOF 3×3×5 supercell used for molecular dynamics simulations.

Supplementary Table 1. The elemental analysis results for SCU-8.

Name	Wight. [mg]	Date Time	Content [%]	Peak Area	Daily Factor
SCU-8	2.9290	18.03.16	N: 1.913	2060	0.9626
			C: 30.29	22981	0.9640
			H: 2.879	6851	0.9844

Supplementary Table 2 Kinetic Parameters of the Pseudo-Second-Order Model for $Cr_2O_7^{2-}$ /MB^{2-}/ ReO_4^- Adsorption on SCU-8. (C_0: Initial concentration; *h*: Initial adsorption rate; *k*: Rate constant; M/V: The solid-to-liquid ratio)

Sorbed anions	C_0 mmol l^{-1}	M/V mmol l^{-1}	pH	Removal %	Second-order kinetic model			
					q_e(mmol mol^{-1})	h(mmol mol^{-1} min^{-1})	k(mol $mmol^{-1}$ min^{-1})	R^2
$Cr_2O_7^{2-}$	0.3	2	5.11	92%	137.36	353.35	0.0187	0.9999
MB^{2-}	0.02	2	6.77	98%	9.121	2.5363	0.0304	0.9987
ReO_4^-	0.04	2	6.15	82%	15.853	471.69	1.877	0.9999

Supplementary Table 3 Parameters of Langmuir and Freundlich isotherms for adsorption of PFOS/ReO_4^-.

	Adsorption isotherm	Parametes	R^2
ReO_4^-	Freundlich $q_e = K_F \times C_e^{1/n}$	n=1.864 K_F=713.2	0.9642
	Langmuir $q_e = \frac{q_m K_L C_e}{1 + K_L C_e}$	K_L=7.5731 q_m=534.07 mmol mol^{-1}	0.9929
PFOS	Freundlich $q_e = K_F \times C_e^{1/n}$	n=1.83 K_F=819.31	0.9000
	Langmuir $q_e = \frac{q_m K_L C_e}{1 + K_L C_e}$	K_L=86.58 q_m=162.08 mmol mol^{-1}	0.9605

Supplementary Table 4 Comparison of adsorption kinetics of PFOS on different adsorbents. (M/V The solid-to-liquid ratio; C_0 Initial concentration; *h* Initial adsorption rate; *k* rate constant;)

Adsorbents	C_0 (mg l^{-1})	M/V mmol l^{-1}	pH	t_e (min)	h (mmol mol^{-1} min^{-1})	*k* (mol $mmol^{-1}$ min^{-1})
SCU-8	1	5(Cl^-)	6.3	10	6.20	8.35
LDHs	1	5(NO_3^-)	6.3	10	4.17	5.67
IRA67	1	5(Cl^-)	6.3	>120	0.46	0.68
PAC	1	5	6.3	60	0.70	0.93
Zeolite (Na-Y)	1	5	6.3	>120	0.06	0.02

Supplementary Table 5 Crystallographic data for SCU-8.

Sample	SCU-8
Formula	$C_{15}H_7O_{7.59}Th$
Mr [g mol^{-1}]	540.74
Crystal system	Hexagonal
Space group	$P6_3/m$
a (Å)	26.009(4)
b (Å)	26.009(4)
c (Å)	9.8065(17)
α	90.00
β	90.00
γ	120.00
V (Å^3)	5745(2)
Z	6
D_c (g cm^{-3})	0.938
μ (mm^{-1})	3.908
F (000)	1486
T(K)	173
GOF on F^2	1.103
R1,[a]wR2[b] (I>2σ(I))	0.0764,0.1895
R1,[a] wR2[b] (all data)	0.0777,0.1903

$^aR_1 = \sum(F_o - F_c)/\sum F_o; wR_2 = [\sum w(F_o^2 - F_c^2)^2/\sum w(F_o^2)^2]^{1/2}$

Supplementary Table 6 Selected bond lengths (Å) for SCU-8

Selected Bond Lengths (Å)			
Th1-O5	2.2684(7)	Th1-O1	2.525(11)
Th1-O1A	2.525(11)	Th1-O1B	2.8843(4)
Th1-O1C	2.8843(4)	Th1-O2	2.481(9)
Th1-O2A	2.481(9)	Th1-O3D	2.499(17)
Th1-O6\O7 (H_2O)	2.83(2)\2.72(4)	Th1-O4D	2.499(17)

Symmetry codes: A=*x, y, 1.5-z*; B=*1-y, x-y, 1.5-z*; C =*1-y, x−y, z*; D=y, *y-x, 0.5+z;*
