# Supplementary material for: A mesoporous cationic thorium-organic framework that rapidly traps anionic persistent organic pollutants
Source: Nat Commun. 2017 Nov 7;8:1354. doi: 10.1038/s41467-017-01208-w (PMC5677036; doi:10.1038/s41467-017-01208-w)
Supplement: Supplementary file 2 — Description of Additional Supplementary Files [file 41467_2017_1208_MOESM2_ESM.pdf]

**Description of Additional Supplementary Files**

File Name: Supplementary Data 1
Description: Cif file for title compound SCU-8.
